# Aging reduces liver resiliency by dysregulating Hedgehog signaling

**DOI:** 10.1111/acel.13530

**Published:** 2022-01-04

**Authors:** Raquel Maeso‐Díaz, George D. Dalton, Sehhoon Oh, Kuo Du, Linda Tang, Tianyi Chen, Rajesh K. Dutta, Jessica H. Hartman, Joel N. Meyer, Anna Mae Diehl

**Affiliations:** ^1^ Division of Gastroenterology Department of Medicine Duke University Health System Durham North Carolina USA; ^2^ Nicholas School of the Environment Duke University Durham North Carolina USA

**Keywords:** aging, Hedgehog, hepatocytes, liver regeneration, resiliency, smoothened

## Abstract

Older age is a major risk factor for damage to many tissues, including liver. Aging undermines resiliency and impairs liver regeneration. The mechanisms whereby aging reduces resiliency are poorly understood. Hedgehog is a signaling pathway with critical mitogenic and morphogenic functions during development. Recent studies indicate that Hedgehog regulates metabolic homeostasis in adult liver. The present study evaluates the hypothesis that Hedgehog signaling becomes dysregulated in hepatocytes during aging, resulting in decreased resiliency and therefore, impaired regeneration and enhanced vulnerability to damage. Partial hepatectomy (PH) was performed on young and old wild‐type mice and Smoothened (Smo)‐floxed mice treated with viral vectors to conditionally delete Smo and disrupt Hedgehog signaling specifically in hepatocytes. Changes in signaling were correlated with changes in regenerative responses and compared among groups. Old livers had fewer hepatocytes proliferating after PH. RNA sequencing identified Hedgehog as a top downregulated pathway in old hepatocytes before and after the regenerative challenge. Deleting Smo in young hepatocytes before PH prevented Hedgehog pathway activation after PH and inhibited regeneration. Gene Ontogeny analysis demonstrated that both old and Smo‐deleted young hepatocytes had activation of pathways involved in innate immune responses and suppression of several signaling pathways that control liver growth and metabolism. Hedgehog inhibition promoted telomere shortening and mitochondrial dysfunction in hepatocytes, consequences of aging that promote inflammation and impair tissue growth and metabolic homeostasis. Hedgehog signaling is dysregulated in old hepatocytes. This accelerates aging, resulting in decreased resiliency and therefore, impaired liver regeneration and enhanced vulnerability to damage.

## Graphical abstract

It is not well understood why older age is a major risk factor for damage to many tissues, including liver. Aging‐related impairments in regeneration may be involved as this limits recovery from injury. Our studies identify a mechanism that explains why aging inhibits liver regeneration by demonstrating that the activity of Hedgehog, a signaling pathway that is necessary for liver growth, is reduced in old hepatocytes.

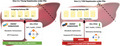

**Abbreviations**
ADP: adenosine diphosphate
BCL2: B‐Cell Lymphoma 2
FCCP: Carbonyl cyanide 4‐(trifluoromethoxy) phenylhydrazone
FOXM1: Forkhead Box Protein M1
Gli: GLI Familiy Zinc Finger
GSEA: Genome Set Enrichment Analysis
HHIP: Hedgehog Intereacting Protein: Angpt, Angiopoietin
IGF: Insulin‐like Growth Factor
IHC: Immunohistochemistry
Ihh: Indian Hedgehog
IL: Interleukin
Mt‐16S: mitochondrial Ribosomal protein S16
Mt‐ND1: mitochondrial NADH dehydrogenase 1
mTOR: mammalian target of rapamycin
PH: Partial Hepatectomy
Ptch: Patched
PTCH: patched
S9: Ribosomal Protein S9
Shh: Sonic Hedgehog
Smo: Smoothened, Frizzled Class Receptor
TERT: telomerase
TNF: Tumor Necrosis Factor

## 1 INTRODUCTION

Older age is a major risk factor for damage to many tissues, including liver (Kennedy et al., 2014). Aging undermines resiliency, i.e., the ability to withstand, and recover from, stressors (López‐Otín et al., 2013). Age‐related deterioration of liver resiliency is important because this both enhances vulnerability to acute and chronic liver injury and causes defective regenerative responses that promote progressive replacement of functional hepatic parenchyma with scar (i.e., cirrhosis; Frith et al., 2009; Poynard et al., 2001; Stine et al., 2013; Thabut et al., 2006). Indeed, similar to other age‐related degenerative diseases, the incidence and prevalence of cirrhosis and potentially lethal complications of cirrhosis, such as liver failure and liver cancer, are increasing as populations age (Maeso‐Díaz & Gracia‐Sancho, 2020). The underlying mechanisms that suppress resiliency during aging are not well understood and thus, therapies that restore tissue resiliency to a more robust, youthful state remain elusive (Hunt et al., 2019; Pibiri, 2018). Preclinical models that challenge resiliency by imposing a regenerative stress are useful for revealing age‐related differences in signaling that associate with reduced resiliency and resultant tissue degeneration. Prior work by us and others demonstrated that aging inhibits liver regeneration after 70% partial hepatectomy (PH) (Nevzorova et al., 2015; Oh et al., 2018; Timchenko, 2009; Timchenko et al., 1998). Therefore, in the present study we used the mouse 70% partial hepatectomy (PH) model to identify age‐related differences in signaling pathways that control liver resiliency.

In the present study, deep sequencing and transcriptomic analysis of RNA isolated from hepatocytes before and after this regenerative challenge identified Hedgehog as one of the signaling pathways that was most differentially activated after PH in young versus old hepatocytes. This discovery has intriguing implications for aging because enforcing Hedgehog signaling was recently reported to extend healthy life span in adult flies (Rallis et al., 2020). Previously, we had shown that inhibiting Hedgehog pathway activity systemically, or selectively in liver stromal cells, of young adult mice blocks liver regeneration after PH (Ochoa et al., 2010; Swiderska‐Syn et al., 2014, 2016). However, more research is necessary to determine if and how activation of this pathway in adult hepatocytes promotes hepatic resiliency because other work by ourselves and others clearly indicates that excessive Hedgehog activity in hepatic stromal cells promotes the evolution of cirrhosis, and shows that uncontrolled Hedgehog signaling in hepatocytes themselves drives hepatocarcinogenesis (Chan et al., 2014; Jung et al., 2007; Verdelho Machado & Diehl, 2018). Therefore, the present study evaluates the provocative hypothesis that Hedgehog signaling becomes deficient in hepatocytes during aging, resulting in decreased hepatocyte resiliency, impaired liver regeneration and therefore, enhanced vulnerability to liver damage. Herein we report definitive evidence that Hedgehog signaling in adult hepatocytes is required to maintain liver resiliency, identify Hedgehog‐sensitive mechanisms that promote resiliency, and show that disrupting Hedgehog signaling in young hepatocytes rapidly phenocopies old hepatocytes, thereby identifying a novel mechanism that helps to explain why aging decreases resiliency and enhances vulnerability to liver damage.

## 2 RESULTS

### 2.1 Liver regeneration is impaired with aging

**FIGURE 1:**
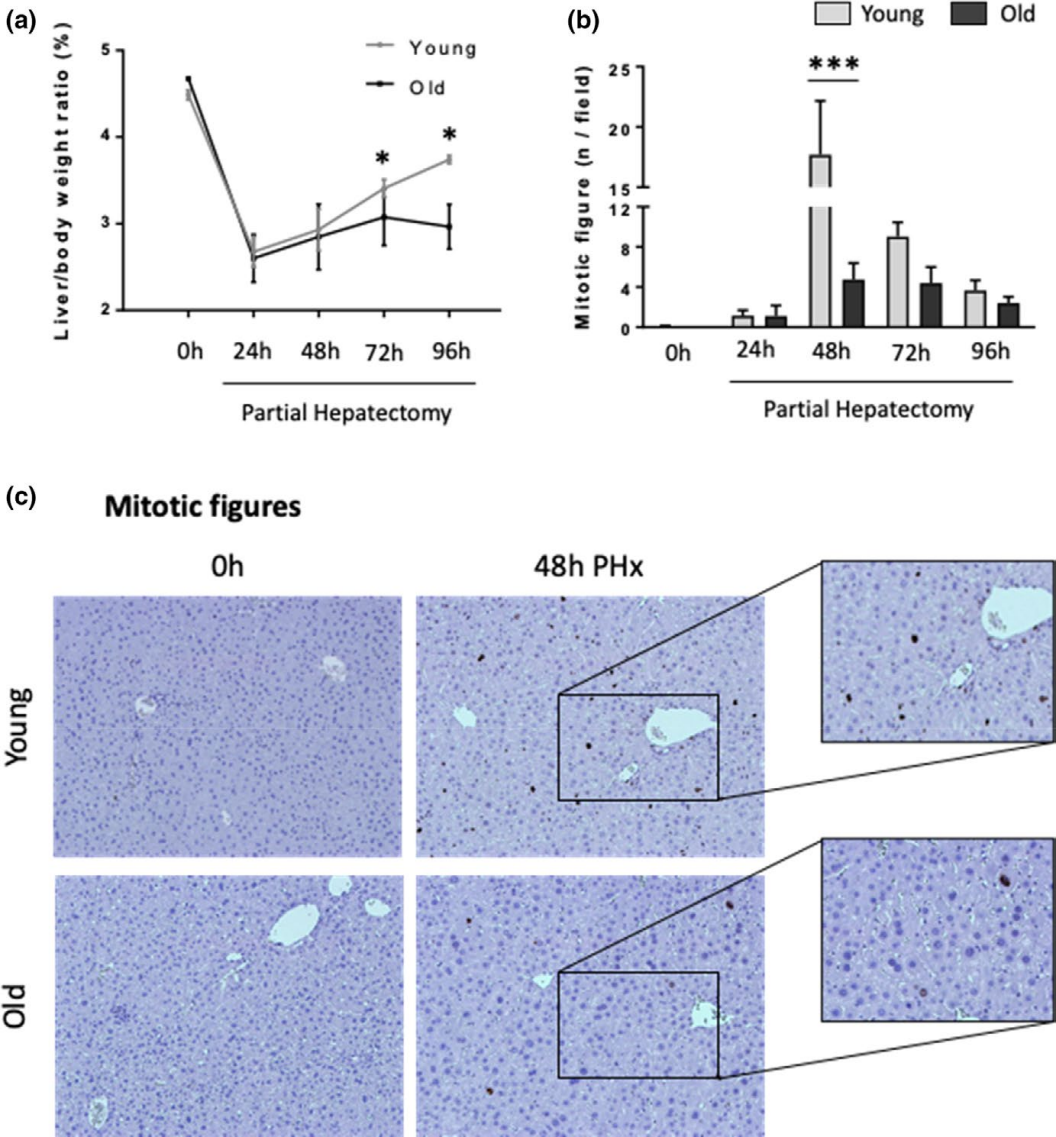
Liver regeneration is impaired with aging. Partial hepatectomy (70%) was performed in young (3‐month‐old) and old (24‐month‐old) mice. Liver tissue and hepatocytes samples were collected at 0 h and 24, 48, 72 and 96 post‐PH. Liver/body weight ratio (a), number of mitotic figures (P‐Histone‐3 positive Hepatocytes) (b) and representative micrographs of P‐Histone 3 immunohistochemistry (c) in liver sections from 0h and 48h following PH in young and old mice (100X magnification). Results shown as MEAN +/− SEM (*n* = 6 mice/group/time, *p < 0.05,***p < 0.001)

Comparison of young and old wild‐type male mice before and at various time points after PH (Figure S1A) confirmed earlier work (Timchenko, 2009) which showed that aging inhibits liver regeneration. Recovery of pre‐PH liver mass was suppressed in old mice relative to young mice (Figure 1a). In young mice, liver weight started to increase 48h after PH, following the peak in hepatocyte mitosis at that time point (Figure 1b, c, Figure S1B and C). The number of hepatocytes entering mitosis after PH was significantly decreased in old mice, suggesting that the reduced proliferative activity of old hepatocytes slowed liver mass recovery after PH (Figure 1b, c, Figure S1B and C).

### 2.2 Hedgehog signaling pathway is downregulated in old and old regenerating hepatocytes

**FIGURE 2:**
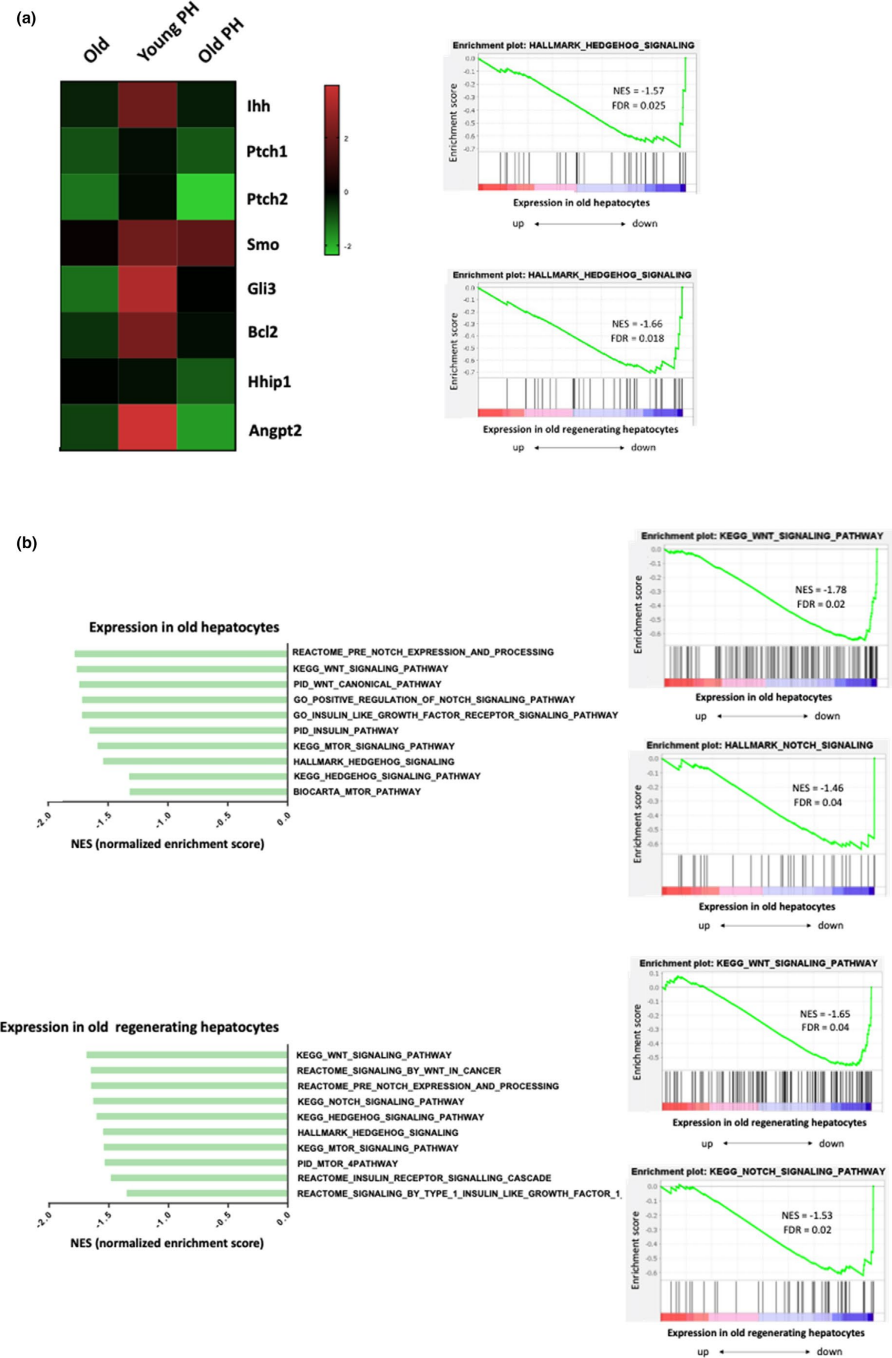
Hedgehog signaling pathway is downregulated in old and old regenerating hepatocytes. RNAseq was performed in hepatocytes isolated from 0 and 48 h post‐PH in young and old mice (*n* = 3 mice/group/time). (a) *Left*, heatmap displaying log2 fold change of signficantly altered Hedgehog pathway genes in old, young regenerating and old regenerating hepatocytes normalized to young hepatocytes. *Right*, gene set enrichment analysis (GSEA) of Hedgehog signaling hallmark signature in old (*top*) and old regenerating (*bottom*) hepatocytes. (b) *Left*, graph displaying normalized enrichment score (NES) of signicantly downregulated Hedgehog target pathways in old (*top*) and old regenerating (*bottom*) hepatocytes normalized to young hepatocytes. *Right*, gene set enrichment analysis (GSEA) of Hedgehog target pathways: Wnt and NOTCH signatures in old (*top*) and old regenerating hepatocytes (*bottom*)

To identify mechanisms for the reduced proliferative response in old livers, we used genome set enrichment analysis (GSEA) and *Deseq* to compare the transcriptomes of young and old hepatocytes before PH and young and old regenerating hepatocytes at 48 h post‐PH. We included only the *Hallmarks* database, defined a very restrictive FDR cutoff (<0.05) for the exploratory GSEA, and discovered that the Hedgehog signaling pathway was one of the most downregulated pathways in both old hepatocytes and old regenerating hepatocytes relative to young hepatocytes (Figure 2a). More detailed *deseq* analysis for genes involved in the Hedgehog pathway confirmed that many Hedgehog‐related genes were significantly downregulated in old hepatocytes including Hedgehog pathway ligands (Indian Hedgehog (Ihh)), receptors (Patched (Ptch)), effectors (Smo and GLI Familiy Zinc Finger 3 (Gli3)), and target genes (B‐Cell Lymphoma 2 (BCL2), Hedgehog Interacting Protein (HHIP) and Angiopoietin (Angpt). Comparison of differentially expressed genes in young and young regenerating hepatocytes also revealed that Hedgehog pathway activity increases in young hepatocytes following a regenerative challenge. Together, these findings provide novel evidence that Hedgehog signaling is suppressed in hepatocytes of old livers and cannot be induced appropriately after liver injury. In a second GSEA, we expanded the number of databases included and assessed other developmental and metabolic pathways that are known to be regulated by Hedgehog. We found that several of these pathways, including Notch, Wnt‐β‐Catenin, insulin like growth factor (IGF), and mammalian target of rapamycin (mTOR)), were significantly downregulated in old and old regenerating hepatocytes (Figure 2b).

**FIGURE 3:**
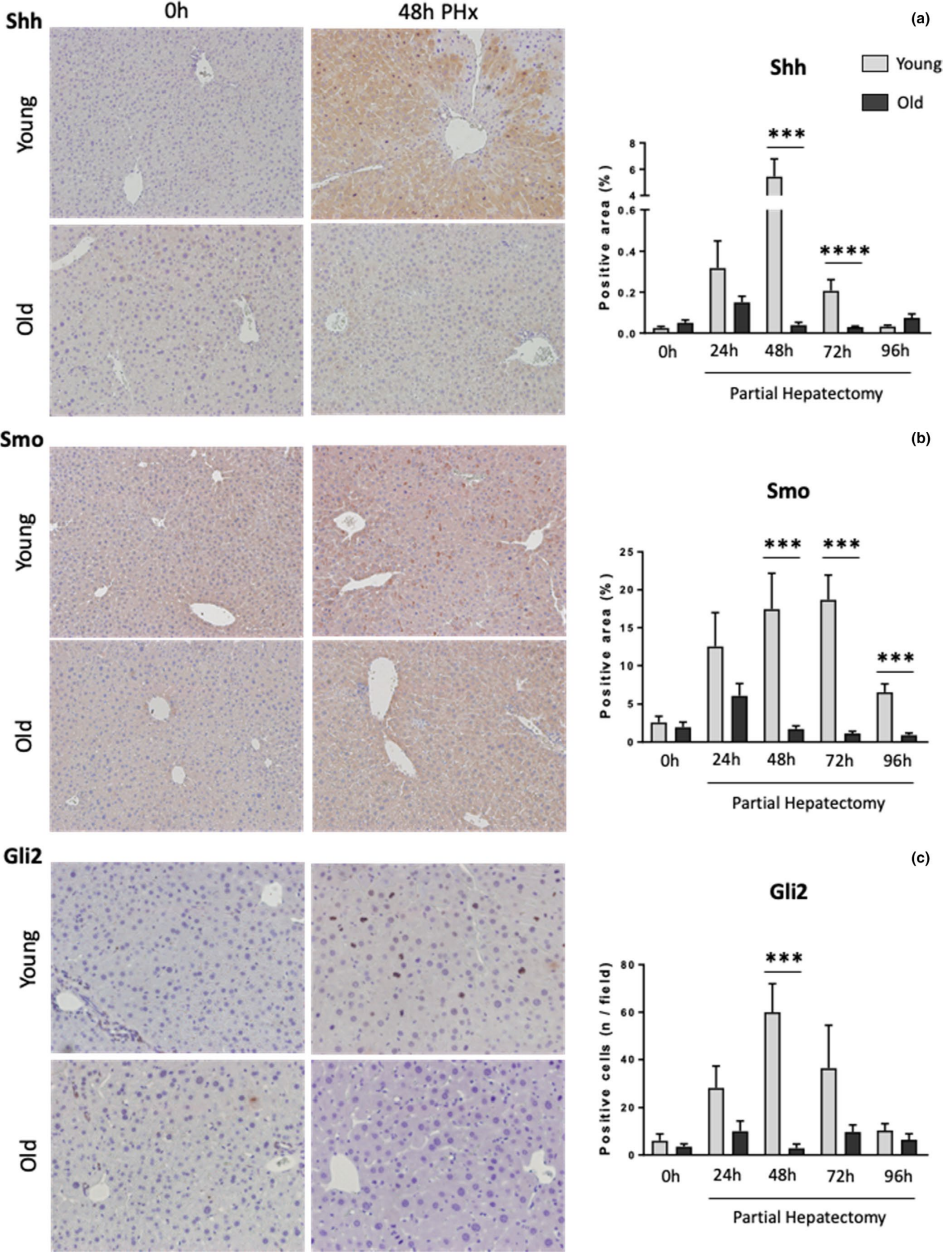
Hedgehog signaling pathway proteins are downregulated in old regenerating hepatocytes. Representative micrographs of Sonic Hedgehog (Shh) (a), Smoothened (Smo) (b) and GLI Family Zinc Finger 2 (Gli2) (c) immunohistochemistry and their corresponding quantifications in liver sections from 0h and 48h following PH in young and old mice (100X magnification). Results shown as MEAN +/− SEM (*n* = 6 mice/group/time, ***p < 0.001, ****p < 0.0001)

To confirm the results derived from the RNA analyses we performed immunohistochemistry (IHC) for proteins in the Hedgehog pathway (Figure 3a–c and Figure S2 and S3). In young livers, mRNAs and protein levels of Ihh and Sonic hedgehog (Shh), another Hedgehog ligand, were very low before PH. However, after PH expression of these mRNAs and proteins increased dramatically and they became widely abundant by 48–72 h post‐PH; thereafter, Hedgehog ligand expression declined rapidly and protein levels were back to normal by 96 h. Similar changes in Hedgehog ligand expression were not observed in the old livers after PH. Binding of Hedgehog ligands to Ptch results in activation of Smo and triggers post‐translational modifications of Gli‐family transcription factors that enable them to localize in nuclei and regulate expression of Hedgehog target genes. Smo also accumulated transiently in subpopulations of hepatocytes in young livers after PH and localized in the cell membranes of zone 2 hepatocytes by 48h after PH. This change in the expression and localization of Smo protein also did not occur in the old livers. Gli2 mainly localized in the nuclei of rare bile duct cells in young livers before PH. 48h after PH, there was a significant increase in the number of hepatocytes expressing Gli2 in the nuclei of young hepatocytes, many of which were undergoing mitosis. However, Gli2 protein could not be detected in the nuclei of old hepatocytes before or after the PH, and the number of mitotic hepatocytes was also reduced significantly in old livers. Therefore, the immunostaining data confirm the mRNA findings and together, demonstrate that hepatocytes in old livers with reduced regenerative capability cannot upregulate the Hedgehog pathway appropriately after PH, suggesting that Hedgehog induction is required to increase hepatocyte proliferation after PH.

### 2.3 Deletion of Smo in young hepatocytes prevents Hedgehog pathway activation after PH

**FIGURE 4:**
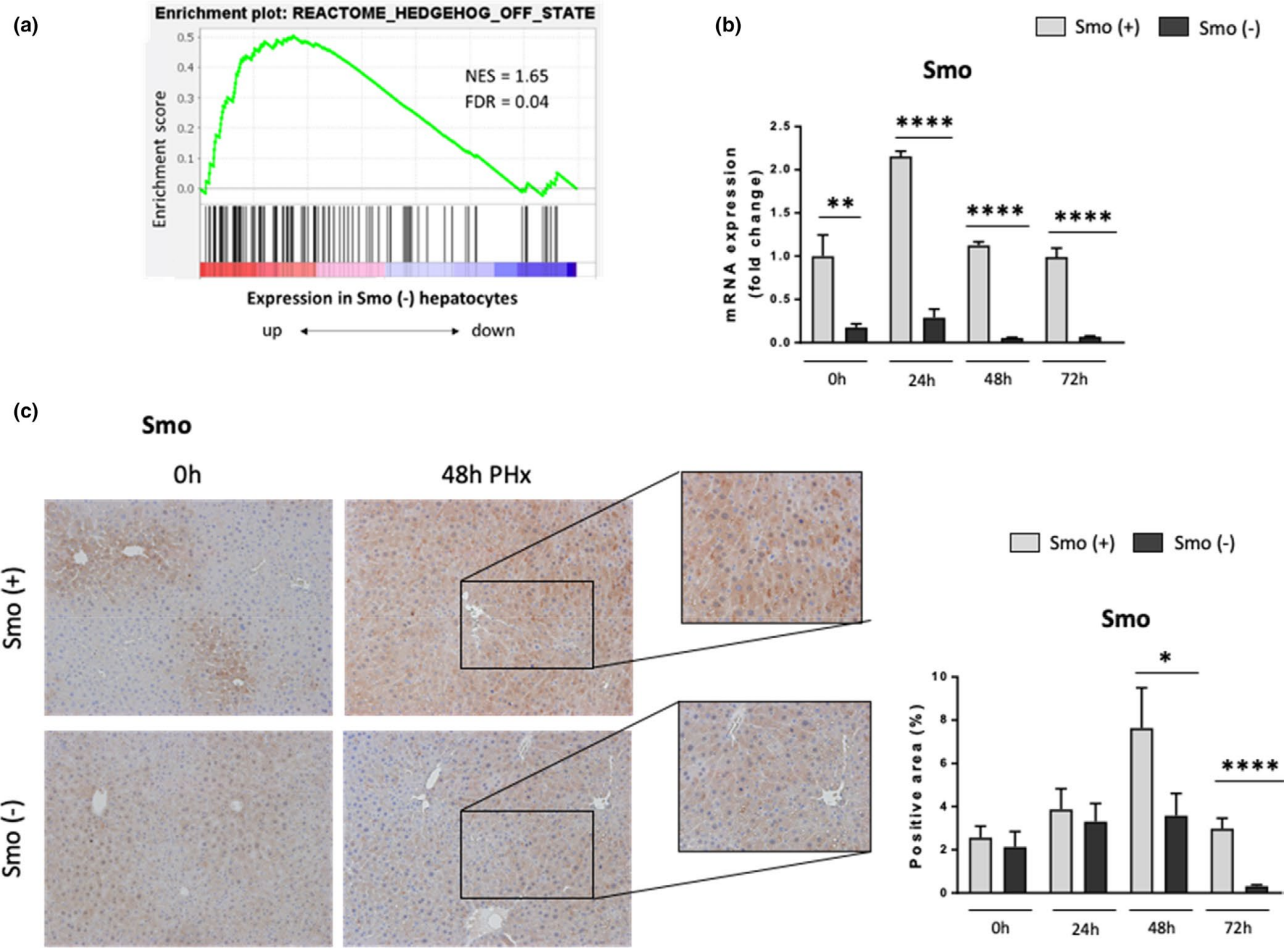
Smoothened deletion in hepatocytes effectively inhibits Hedgehog signaling pathway. Smo‐floxed mice were injected with AAV8‐TBG‐Cre recombinase (Smo (−) or AAV8‐TBG‐Luciferase (Smo (+). Partial hepatecetomy (PH) was performed seven days later. Liver and hepatocytes were harvested before or 24, 48, 72 and 96h post‐PH. GSEA of Hedgehog‐off‐state signaling signature in Smo (−) hepatocytes (a). Smoothened (Smo) hepatocyte mRNA expression (b). *Left*, representative micrographs and *Right*, quantitative analysis of Smo immunohistochemistry in liver sections (100X magnification) (c). Results shown as MEAN +/− SEM (*n* = 5 mice/group/time, *p < 0.05, **p < 0.01, ***p < 0.001, ****p < 0.0001)

Previously, we reported that induction of hepatocyte proliferation after PH required activation Hedgehog signaling in myofibroblastic cells (Swiderska‐Syn et al., 2014), consistent with evidence that these stromal cells are critical sources of hepatocyte mitogens and other growth factors. However, our new findings in old mice link suppression of Hedgehog signaling in the hepatocytes themselves with reduced hepatocyte proliferation post‐PH. Therefore, to determine if decreased Hedgehog signaling in hepatocytes might be responsible for suppressing regenerative responses in old livers, we used viral vectors to conditionally delete Smo specifically in hepatocytes of young Smo‐floxed male mice and compared Hedgehog pathway activation and hepatocyte proliferation in Smo (−) and Smo (+) mice at various time points after PH (Figure S4A). Treatment of young Smo‐floxed mice with the Cre vector significantly reduced Smo mRNA expression before PH (time 0) and after PH (Figure 4a). Smo protein detection by IHC confirmed that Smo was expressed in hepatocyte cell membranes in Smo (+) mice at 48h after PH, but not in Smo (−) mice (Figure 4b and Figure S4C). Importantly, GSEA of hepatocyte RNAseq data demonstrated that deleting Smo significantly upregulated the Hedgehog‐OFF‐state signature (Figure 4a), proving that Hedgehog signaling was disrupted. Consistent with this, IHC showed that Smo deletion reduced the numbers of hepatocytes with Smo(+) at 48h post‐PH (Figure 4b, c). In addition, qRT‐PCR demonstrated that Smo deletion inhibited upregulation of Gli2 mRNA in hepatocytes post‐PH (Figure S4D). Induction of Ihh mRNA and protein expression was also inhibited after PH in the Smo (‐) group (Figure S4D).

### 2.4 Deletion of Smo in hepatocytes inhibits hepatocyte proliferation after partial hepatectomy

**FIGURE 5:**
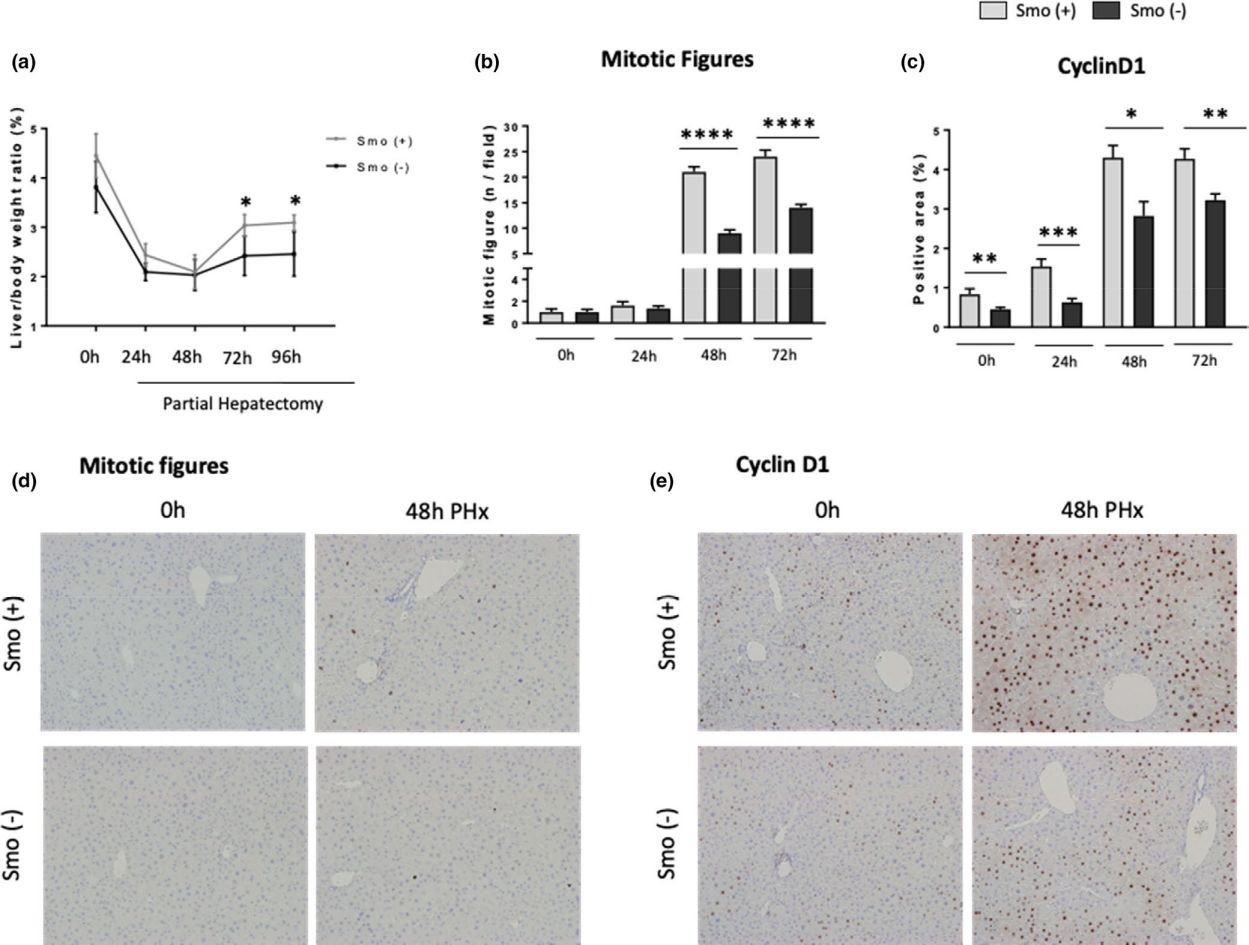
Smoothened deletion in hepatocytes inhibits liver regeneration after partial hepatectomy. Liver to body weight ratios (a), quantifications and their representative micrographs of mitotic figures (b, d) and Cyclin D1 (c, e) immunohistochemistry in liver sections from 0 and 48 h following PH in Smo (+) and Smo (−) mice (100× magnification). Results shown as MEAN +/− SEM (*n* = 5 mice/group/time, *p < 0.05, **p < 0.01, ***p < 0.001, ****p < 0.0001)

Liver regeneration was inhibited in mice with Smo‐deleted hepatocytes as evidenced by reduced restoration of liver‐to‐body weight ratios after PH (Figure 5a). As in old mice (Figure 2), suppressed recovery of liver mass in young mice with reduced Hedgehog pathway activity reflected decreased hepatocyte proliferative activity. Compared to Smo (+) mice, Smo (−) mice had fewer hepatocytes in mitosis after PH (Figure 5b,d and Figure S5E) and exhibited reduced expression of cyclin D1 (Figure 5c, e and Figure S5B, F), a cell cycle protein that controls transition through G1‐S and is necessary to increase hepatocyte proliferation after PH (Leong et al., 1964).

Hedgehog signaling also induces cellular proliferation by upregulating FOXM1, another cell cycle regulator that is required for hepatocyte proliferation after PH (Xiang et al., 2014). Transcript levels of FOXM1 and Ki67, a protein widely used as a proliferation marker, were also significantly downregulated in the Smo‐deficient mice (Figure S5A, 5C and 5D). Together, these results confirm that deleting Smo disrupts Hedgehog signaling in adult hepatocytes and prove that simply abrogating Smo activity in these cells is sufficient to prevent them from progressing through the cell cycle to regenerate the liver after PH.

### 2.5 Smo (‐) hepatocytes resemble aged hepatocytes

Having discovered that old hepatocytes (which are known to have reduced proliferative activity) have impaired Hedgehog signaling and proven that simply disrupting Hedgehog signaling in young hepatocytes is sufficient to inhibit their proliferation, we next sought to identify differentially regulated pathways that could account for this shared ‘reduced regenerative’ phenotype. Bulk RNAseq was performed in hepatocytes isolated from Smo (+) or Smo (−) mice. Genes that were significantly upregulated or downregulated in Smo (−) hepatocytes relative to control Smo (+) hepatocytes were filtered and compared to the list of genes that were differentially expressed in old hepatocytes compared to control young hepatocytes. Old and Smo (−) hepatocytes shared 468 significantly upregulated genes and 333 significantly downregulated genes (Figure S6A). The number of similarly regulated genes was significantly greater than would be expected by chance alone (*p* = 1.06 × 10^−53^ for upregulated genes and *p* = 1.81 × 10^−05^ for downregulated genes). GSEA and Gene Ontology analysis revealed that both old and Smo (−) hepatocytes were significantly depleted of transcripts involved in ‘cellular component biogenesis’ and ‘morphogenesis of an epithelium’ and exhibited suppressed activities of several growth factor signaling pathways, including insulin, IGF, EGF, HGF/Met, Wnt/beta catenin, and NOTCH. Cellular processes that critically orchestrate the proliferative state were also significantly repressed in both old and Smo‐depleted hepatocytes. Examples included the MAPK pathway, Ras protein signal transduction, mitotic prophase, regulation of cell cycle progression, meiotic synapsis, Rho A pathway, regulation of cell shape, cellular component maintenance, chromatin remodeling. Pathways that coordinate cell metabolism with changes in energy supply and demand, such as circadian regulation of gene expression, amino acid metabolic process, acyl CoA metabolic process, cellular response to oxygen levels, response to starvation, mitochondrial biogenesis, were also significantly downregulated in both old and Smo (−) hepatocytes. In contrast, pathways that were commonly *up*regulated in young Smo‐depleted hepatocytes and old wild‐type hepatocytes included those that promote sterol and lipid accumulation, such as reverse cholesterol transport, positive regulation of cholesterol esterification, positive regulation of fatty acid biosynthetic process, and triglyceride metabolic process. Other *up*regulated pathways in old and Smo (−) hepatocytes control cellular stress and viability, such as regulation of superoxide metabolic process, glutamate and glutathione metabolic process, cellular detoxification, cellular response to hypoxia, iron ion homeostasis, regulation of mitochondrial membrane potential, regulation of intrinsic apoptotic signaling pathway. After pathways involved in regulation of lipid homeostasis, pathways for ‘innate immunity/antimicrobial defense’ were the second most upregulated processes in old hepatocytes and Smo‐depleted young hepatocytes according to GSEA. This was unexpected because our GSEA was performed on RNA isolated from hepatocytes. Nevertheless, the findings were confirmed by the robust Gene Ontogeny results which showed that relative to their respective controls, old hepatocytes and Smo (−) hepatocytes were enriched for numerous processes the promote cytokine/chemokine activity. Examples include regulation of interleukin (IL)1 production, positive regulation of tumor necrosis factor (TNF) production, regulation of IL6 production, cellular response to interferon‐gamma, acute phase response. Pathways involved in immune cell responses were also excessively induced, such as positive regulation of T cell mediated cytotoxicity, neutrophil activation involved in immune response, granulocyte chemotaxis, regulation of phagocytosis, defense response to bacterium, defense response to fungi. Tables S2–S7 list the top 20 differentially upregulated and downregulated pathways in old versus young hepatocytes and Smo (−) versus Smo (+) hepatocytes. Together, the transcriptomic analyses indicate that deleting Smo in young hepatocytes is sufficient to phenocopy the growth‐inhibited, metabolism‐defective, pro‐inflammatory gene expression profile of old hepatocytes, suggesting that reduced resiliency in old hepatocytes associates with a pro‐inflammatory state caused by decreased Smo activity.

**FIGURE 6:**
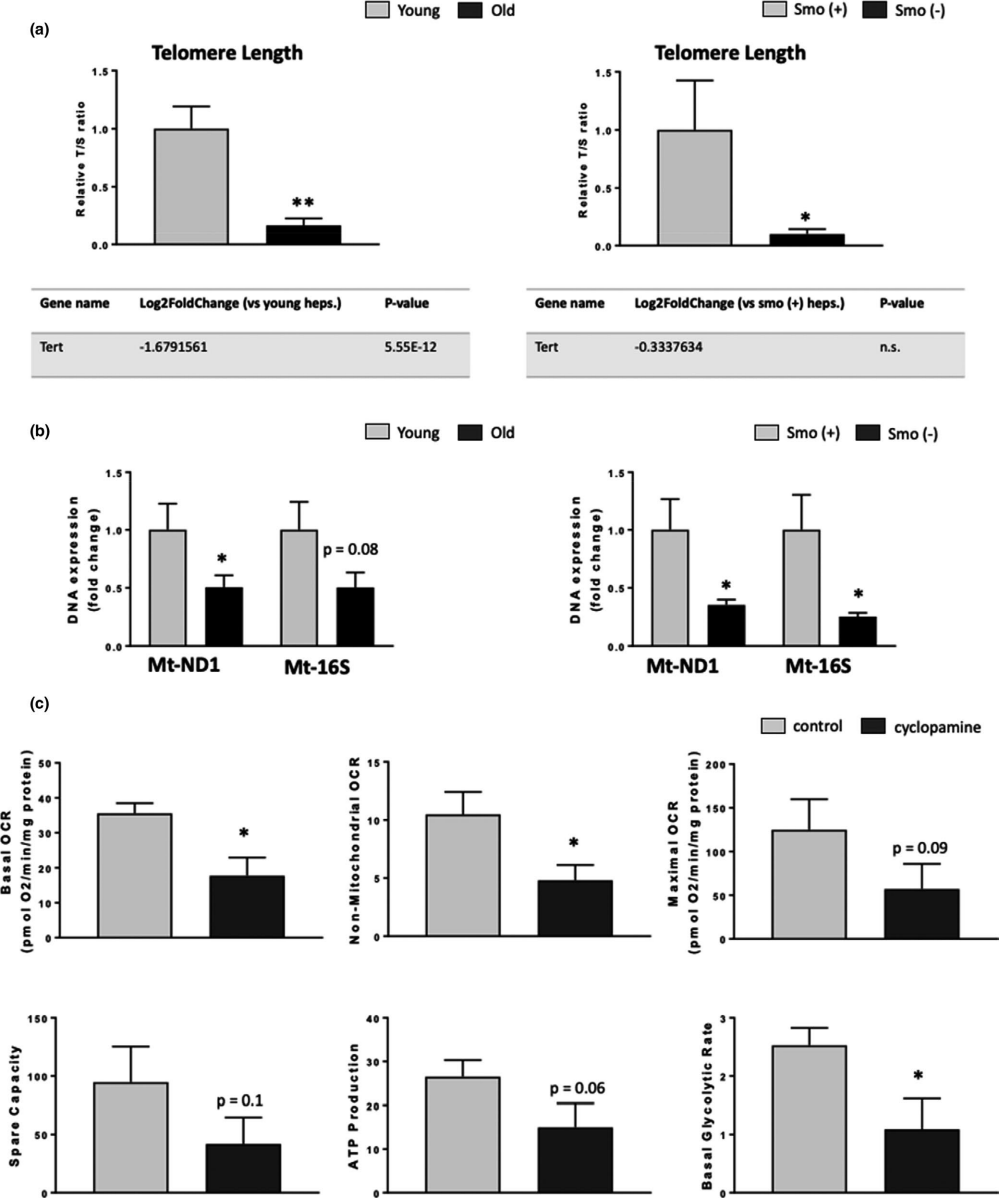
Smoothened deficient young hepatocytes resemble old hepatocytes. (a) Telomere length (*top*), Tert (*bottom*) and (b) mitochondrial ND1 and 16S relative gDNA expressions from young and old (*Left*) and Smo (+) and Smo (−) (*Right*) mice. Results shown as MEAN +/− SEM (*n* = 5 mice/group/time, *p < 0.05, **p < 0.01). Primary hepatocytes isolated from healthy control mice were treated for 24 h with vehicle (DMSO) or 5 μM Cyclopamine (*n* = 4 mice/group). Mitochondrial bioenergetics were assessed using a Seahorse XF96 analyzer after the incubation time. (c) Basal Oxygen Consumption Rate (OCR), non‐mitochondrial OCR, maximal OCR, spare capacity, ATP‐linked oxygen consumption and basal glycolytic rate in hepatocytes treated with vehicle or cyclopamine. Results shown as MEAN +/− SEM (*p < 0.05)

To more directly determine whether the lack of Smo in hepatocytes promotes aging‐related loss of resiliency, we manipulated Smo and analyzed effects on two key functional consequences of aging that negatively impact growth and promote progressive tissue degeneration: telomere attrition and mitochondrial dysfunction. Compared to young livers or Smo (+) livers, both old and Smo (−) livers exhibited significant telomere shortening (Figure 6a, b). In old hepatocytes, mRNA expression of telomerase (tert), the enzyme responsible for telomere maintenance, was significantly downregulated whereas telomere shortening occurred despite preservation of telomerase gene expression in young Smo (−) hepatocytes. Telomere shortening is a consequence of DNA damage and chronic inflammation and thus, associates with mitochondrial dysfunction (Fang et al., 2016; Horssen et al., 2019; Jurk et al., 2014). Relative to their respective controls, both Smo (−) young hepatocytes and old hepatocytes also exhibited decreased hepatocyte mitochondrial DNA copy number, as assessed with two important mitochondrially encoded genes; NADH dehydrogenase 1 (MT‐ND1) and ribosomal protein S16 (MT‐S16), suggesting that the number of mitochondria in these livers was reduced (Figure 6b). Mitochondrial loss is predicted to compromise energy homeostasis and reducing energy stores is known to restrict growth (Khiati et al., 2015). Therefore, we treated young primary hepatocytes with cyclopamine, a direct Smo antagonist that was shown to inhibit liver regeneration after PH (Ochoa et al., 2010) or vehicle for 24 h and performed a Seahorse study to determine how disrupting Hedgehog signaling acutely impacted mitochondrial respiration (Figure 6c and Figure S6B). Inhibiting Smo rapidly suppressed basal and maximal respiration, and reduced capacity for ATP synthesis in hepatocytes, demonstrating that hepatocytes require Smo activity to maintain mitochondrial function. Together, the findings prove that young hepatocytes require Smo activity to remain resilient and show that old hepatocytes phenocopy non‐resilient, Smo‐deficient young hepatocytes.

## 3 DISCUSSION

Loss of resiliency occurs during aging and limits the ability of tissues to tolerate and recover from various stresses, leading to gradual degeneration of tissue structure and function. Consistent with this, aging enhances vulnerability to acute liver injury and compromises repair, thereby promoting chronic liver damage. Thus, it is not surprising that older age is a major risk factor for decompensated cirrhosis, the fatal end stage of all types of chronic liver disease. It follows that age‐related increases in the incidence and prevalence of cirrhosis and consequent hepatic decompensation could be reduced by restoring hepatic resiliency. However, more research to identify tractable mechanisms that undermine liver resiliency is required in order to achieve this objective.

Herein we report novel evidence that Hedgehog signaling is suppressed in old hepatocytes and show that this helps to explain how aging impairs hepatocyte resiliency by demonstrating that targeted disruption of the Hedgehog pathway in young hepatocytes immediately phenocopies consequences of aging that reduce hepatocyte resiliency in old hepatocytes. More specifically, hepatocyte‐specific deletion of Smo, an obligatory upstream component of the Hedgehog signaling pathway, abrogates downstream signaling that is known to enable proliferation in many cell types, including induction of critical drivers of cell cycle progression such as cyclin D1, FoxM1 and Ki67. As in old hepatocytes, loss of Smo activity in young hepatocytes curtails increases in hepatocyte mitosis that must occur to replenish the hepatocyte population and effectively regenerate the liver after PH.

A conserved, Smo‐sensitive mechanism for this reduced hepatocyte proliferative activity was also identified in old hepatocytes and young Smo‐depleted hepatocytes, namely, telomere shortening. Accumulation of hepatocytes with short telomeres associates with liver atrophy and progressive hepatic dysfunction in young humans, and aging enriches the liver with hepatocytes that have shortened telomeres (Wiemann et al., 2002). In mice, defective telomere maintenance in hepatocytes has been linked to an increased risk for chronic inflammatory conditions, and exacerbates cirrhosis induced by carbon tetrachloride (Sato et al., 2004). Interestingly, simply provoking telomere shortening in the intestinal epithelial lineage was shown to cause intestinal inflammation in mice, inducing an inflammasome gene signature in intestinal epithelial cells and increasing their production of IL1, IL‐18, TNFα, and interferon‐gamma proteins, leading to intestinal accumulation of cytotoxic T cells. These responses were accompanied by mild intestinal degeneration in young mice that gradually worsened with age, and was rescued by reactivating telomerase (Chakravarti et al., 2020).

Telomerase restores telomere length after DNA damage, and cell cycle progression is generally blocked until the defective telomeres are repaired (Barnes et al., 2019). When challenged by DNA damaging agents, blood cell lineages that activate Smo are able to escape the checkpoint inhibition that is typically induced by DNA damage and thus, can progress through the cell cycle despite having DNA adducts; blocking Smo activation abrogates DNA damage‐resistant proliferation in those cells (Scheffold et al., 2020). The mechanisms whereby Smo enables resistance to typical sequelae of DNA damage remain poorly understood but Hedgehog‐regulated, Gli‐family transcription factors are known to promote telomerase transcription in some cells (Mazumdar et al., 2013). Our findings indicate that Gli‐activity is reduced in old hepatocytes and show that telomerase mRNA expression is also reduced in these cells, suggesting that repair of shortened telomeres is reduced by inhibiting Hedgehog signaling and renders old hepatocytes susceptible to checkpoint inhibition that arrests their progression through the cell cycle. On the other hand, telomerase mRNA levels were not decreased in young hepatocytes a week or so after Smo was deleted acutely, although these young Smo (−) cells had shortened telomeres and reduced proliferative activity. Cell cycle arrest after DNA damage has been linked with reduced expression of circadian clock genes in blood cell lineages with low Hedgehog pathway activity (Scheffold et al., 2020). Interestingly, our GSEA demonstrated decreased ‘circadian regulation of gene expression’ in Smo‐deficient hepatocytes, even in young mice.

More research is necessary to determine how inhibiting Smo promotes telomere loss and impacts DNA damage responses in hepatocytes. One possibility is that progressive DNA damage and telomere shortening are both by‐products of an inflammatory response in liver tissue that is triggered by dysfunctional telomeres in Smo‐deficient hepatocytes, similar to the tissue inflammation and degeneration that occurred in intestinal mucosa when telomere function was directly disrupted in intestinal epithelial cells (Chakravarti et al., 2020, 2021; Jurk et al., 2014). In this regard, it is important to note that inflammatory cytokines increase mitochondrial membrane permeability and thereby, exacerbate cellular exposure to mitochondria‐derived reactive oxygen species (ROS) that cause oxidative DNA damage (Horssen et al., 2019). Oxidative damage to the nuclear genome, in turn, is known to promote cell cycle arrest and telomere loss (Barnes et al., 2019), responses that perpetuate inflammation and drive further tissue degeneration. We discovered that blocking Smo activity disrupted mitochondrial function, inhibited mitochondrial biogenesis, and reduced mitochondrial mass in hepatocytes. Whether or not the acute mitochondrial sequelae of Smo‐disruption are adaptive and help hepatocytes constrain oxidative stress in an inflammatory hepatic microenvironment requires further study. On the other hand, these responses are predicted to suppress hepatocyte regeneration based on evidence that inhibiting complex I in the mitochondrial electron transport chain, or depleting mitochondrial DNA, cause mitotic aberrations that arrest cells in G2‐M (Donthamsetty et al., 2014).

It is important to emphasize that our study has limitations and raises questions that will require further research to address. First, we did not study female mice in order to minimize potentially confounding effects of aging on sex hormones. However, as Hedgehog signaling and many other aspects of liver biology are sexually di‐morphic, future studies are needed to determine if reduced Hedgehog signaling also contributes to aging‐related decreases in hepatocyte resiliency and liver regenerative capacity in female mice. Second, the hepatocyte RNAseq data presented in this paper were generated by deep sequencing bulk RNA from hepatocyte isolates derived from a relatively small number of Smo‐floxed mice. More studies are needed to confirm the reproducibility of our findings but the bulk RNAseq approach is not ideal because it may obscure zonal differences in Hedgehog pathway activity. This is an important consideration since other morphogenic signaling pathways are zonally localized in healthy adult livers, and both hepatocyte metabolism and proliferative activity are known to vary along the hepatic perfusion gradient (Halpern et al., 2017; MacParland et al., 2018; Ramachandran et al., 2019). To rectify the limitations of our bulk RNAseq analysis, we are currently performing single cell RNA sequencing (scRNA seq) studies of thousands of hepatocytes isolated from additional Smo (+) and Smo (+) mice. This approach can resolve concerns about data reproducibility since it characterizes the transcriptomes of thousands of hepatocytes at the single cell level, and clusters cells according to similarities in their gene expression profiles. The available results from this ongoing work confirm, complement and extend the findings of the bulk RNAseq data about the effects of Smo deletion on hepatocyte resiliency that are reported in the present manuscript. Most importantly, upregulated genes in our Smo‐deleted hepatocytes overlap significantly with upregulated genes in hepatocytes obtained from 30‐month‐old mice that was recently published in a single cell transcriptomic atlas of aging tissues in the mouse (Zhang et al., 2021) (data not shown). Finally, more research is needed to determine if the aging‐related decline in hepatocyte Hedgehog signaling helps to explain why solid epidemiologic evidence demonstrates a decreased incidence of HCC in the very elderly when the incidence of hepatocellular carcinomas (HCC) increases progressively until about 70 years of age (Sheedfar et al., 2013). The authors of a recent review speculate that this paradox might reflect the cumulative effects of age‐related declines in insulin growth factor (IGF)‐1 signaling (Xu et al., 2013). Loss of IGF1 activity inhibits liver regeneration (Desbois‐Mouthon et al., 2006) and thus, is expected to promote tissue degeneration, but it might also curtail the outgrowth of malignant hepatocytes (Adamek & Kasprzak, 2018) and thereby protect against hepatocarcinogenesis. This concept merits further evaluation given evidence that inhibiting Smo activity in hepatocytes disrupts the IGF‐IGFreceptor1 axis (Matz‐Soja et al., 2014).

In conclusion, we have discovered that hepatocytes in old livers have diminished Hedgehog pathway activity and reduced resiliency in response to a regenerative challenge. Further, we have proven that disrupting Smoothened in young hepatocytes abrogates constitutive Hedgehog signaling, rapidly phenocopies the gene expression profile of old hepatocytes, and recapitulates the old hepatocyte regenerative defects. Pathway analysis identified conserved activation of inflammatory signaling and suppression of growth factor signaling in old hepatocytes and Smo‐deleted young hepatocytes. Both old‐ and Smo‐deleted hepatocytes also exhibited telomere attrition and mitochondrial dysfunction, further supporting a shared mechanism for deficient regenerative capacity. Together, these findings identify a novel role for constitutive hepatocyte Hedgehog signaling in maintaining liver resiliency over the life span and suggest that restoring pathway activity in old hepatocytes might improve liver resiliency and thwart aging‐related increases in susceptibility to liver damage.

## 4 EXPERIMENTAL PROCEDURES

### 4.1 Animal experiments

C57BL/6J 3‐month‐old wild‐type (Young) mice were obtained from Jackson Laboratories (Bar Harbor, ME). C57BL/6J 24‐month‐old wild‐type (Old) mice were obtained from the NIA Aged Rodent Colonies (https://www.nia.nih.gov/research/dab/agedrodent‐colonies‐handbook). Adult male, C57BL/6J wild‐type (WT) mice (Jackson Laboratory, Bar Harbor, ME) and adult male Smoothened (Smo)^tm2Amc^/J (Smo‐flox) mice on a C57Bl6/J background were purchased from the Jackson Laboratory (Bar Harbor, ME) and studied at 2–3 months of age. All mice were housed in a barrier facility on 12 h:12 h light cycle with free access to water and standard chow diet (Rodent diet 20, 5033; Picolab, St. Louis, MO). Male Smo flox/flox mice were injected by tail vein with 5 × 10^11^ genome‐equivalents of AAV8‐TBG‐Luc (control; Smo (+); *n* = 50 mice) or AAV8‐TBG‐Cre (Smo (−); *n* = 50 mice) to selectively delete the Smoothened (Smo) gene in hepatocytes. Viruses were obtained from the University of Pennsylvania Viral Vector Core.

70% partial hepatectomy (PH) (Nevzorova et al., 2015; Swiderska‐Syn et al., 2014) was performed on young and old wild‐type mice and young Smo‐floxed mice without or with Smo deletion. Smo‐floxed mice underwent PH 7 days after viral injection based on pilot studies showing reproducibly efficient deletion of floxed Smo alleles by this time point. Mice were sacrificed before PH (0h) and at 24, 48, 72, or 96 h after PH to obtain either liver tissue (60 young and old mice, *n* = 6 mice/group/time point and 50 smo (+) and smo (−) mice, *n* = 5 mice/group/time point) or primary hepatocytes (60 young and old mice, *n* = 6 mice/group/ time point and 50 smo (+) and smo (−) mice, *n* = 5 mice/group/time point). At whole liver harvest, slices of liver were formalin‐fixed for paraffin embedding and the remainder snap frozen in liquid nitrogen for RNA and protein analysis.

Animal care and surgical procedures were conducted in compliance with an approved Duke University IACUC protocol, and those set forth in the “Guide for the Care and Use of Laboratory Animals” as published by the National Research Council.

### 4.2 Hepatocyte isolation

To obtain primary hepatocytes, livers of the remaining mice were perfused with collagenase as described (Oh et al., 2018). Hepatocyte preparations were evaluated by light microscopy to assure that viability and purity were at least 95%. Freshly isolated hepatocytes were immediately processed to obtain RNA and protein.

### 4.3 RNA‐Seq and analysis

Global transcriptome profiling was performed by RNAseq using freshly isolated hepatocytes from young and old wild‐type mice (*n* = 3 mice/group/time point) or Smo‐floxed mice (*n* = 1–2 mice/group/time point) before and at 48 h after PH. Total RNA was extracted using RNeasy mini kit (Qiagen, Hilden, Germany) following the manufacturer's instructions. mRNA library preparation (poly A enrichment) and mRNA‐Seq was performed by Novogene using Illumina NovaSeq PE150 platform. Resulting data was pre‐processed: *Trim_galore* was used to trim off adapter and low‐quality reads, *STAR* for reads alignment to the reference genome (mm10_STAR_genome_idx), *Samtools index* sorted bam files, *Picard* removed duplicates and *HTSeq* for quantification of the gene expression data. Differential expression analysis and gene set enrichment analysis were performed using *Deseq* and *GSEA*, respectively. The dataset is available at the National Center for Biotechnology Information (NCBI) Gene Expression Omnibus database, accession number GSE18176.

### 4.4 Statistics

Data are expressed as mean ± SEM, unless otherwise specified. Statistical significance between two groups was analyzed by two‐tailed Student's *t* test, whereas comparisons of multiple groups were evaluated by two‐way analysis of variance (ANOVA) as specified followed by a post‐hoc Tukey's test. *p*‐values <0.05 were considered statistically significant. Drawing graphs and statistical analyses were performed using GraphPad Prism 8 (GraphPad Software, Inc. La Jolla, CA, USA).

## CONFLICT OF INTEREST

The authors have declared that no conflict of interest exists.

## AUTHOR CONTRIBUTIONS

RMD designed the research studies, conducted experiments, acquired data, analyzed data, provided reagents, wrote the manuscript; GDD, SHO, KD, LT, TC, RKD conducted experiments and acquired data. JHH and JNM designed seahorse experiments and analyzed data. AMD designed the research studies, analyzed data, wrote the manuscript, and secured funding for the research.

## Supporting information

Supplementary Material

## Data Availability

The data supporting the findings of this study are available within the article and/or supplementary materials.
