# Supplementary material for: Aging reduces liver resiliency by dysregulating Hedgehog signaling
Source: Aging Cell. 2022 Jan 4;21(2):e13530. doi: 10.1111/acel.13530 (PMC8844109; doi:10.1111/acel.13530)
Supplement: Supplementary file 1 — Supplementary Material [file ACEL-21-e13530-s001.docx]

**Aging reduces liver resiliency by dysregulating hedgehog signaling**

Raquel Maeso-Díaz ^1^, George D. Dalton ^1^, Sehhoon Oh ^1^, Kuo Du^1^, Linda Tang^1^, Tianyi Chen ^1^, Rajesh K. Dutta ^1^, Jessica H. Hartman ^2^, Joel N. Meyer ^2^ & Anna Mae Diehl ^1^

^1^ Division of Gastroenterology, Department of Medicine, Duke University Health System, Durham, North Carolina, USA.

^2^ Nicholas School of the Environment, Duke University, Durham, North Carolina, USA.

**Table of contents**

Supplementary materials and methods…………………………………………………2

Supplementary table 1………………….…………………………………………………5

Supplementary table 2………………….…………………………………………………6

Supplementary table 3………………….…………………………………………………7

Supplementary table 4………………….…………………………………………………8

Supplementary table 5………………….…………………………………………………9

Supplementary table 6………………….…………………………………………………10

Supplementary table 7………………….…………………………………………………11

Supplementary Figure 1…………………………………………………………………..12

Supplementary Figure 2…………………………………………………………………..14

Supplementary Figure 3…………………………………………………………………..15

Supplementary Figure 4…………………………………………………………………..16

Supplementary Figure 5…………………………………………………………………..18

Supplementary Figure 6…………………………………………………………………..20

Supplementary References………………………………………………………………22

**Supplementary materials and methods**

**Two-step real time RT-PCR**

Total RNA was extracted from primary hepatocytes using RNeasy mini kit (Qiagen, Hilden, Germany) or TRIzol (Invitrogen, Carlsbad, CA) according to the manufacturer’s instructions. The concentration and purity of RNA were determined using a Nanodrop 2000 spectrophotometer. Template complementary DNAs were synthesized from total RNA using the High-Capacity cDNA Reverse Transcription Kit (Applied Biosystems, Foster City, CA) according to the manufacturer's protocols. Power SYBR Green Master Mix (Applied Biosystems) was used for real-time quantitative PCR (qPCR) on the manufacturer’s specifications (StepOnePlus™ Real-Time PCR System, Applied Biosystems). The qPCR results were normalized to the housekeeping gene 40S ribosomal proteins S9 (S9) mRNA based on the threshold cycle (C_t_) and relative fold-change was determined using the 2^-ΔΔCt^ method. The sequences of primers used in this study are listed (Table S1).

**Liver histology and immunohistochemistry**

Liver specimens were fixed in 10% neutral buffered formalin, embedded in paraffin using standard methods and cut into 5 µm sections. For immunohistochemistry, liver sections were deparaffinized, hydrated and incubated in 3% hydrogen peroxide to block endogenous peroxidase. Antigen retrieval was performed by heating in 10 mM sodium citrate buffer (pH 6.0) for 10 min using a microwave. Specimens were blocked in Dako Protein Block solution (Agilent, Santa Clara, CA) for 30 min at room temperature followed by incubation with primary antibody at 4 °C overnight. Immunohistochemistry was used to detect target proteins using the following antibodies: S10P-Histone3 (ab5176, Abcam), Sonic hedgehog (Shh) (ab73958, Abcam), Indian hedgehog (Ihh) (sc-13088, Santa Cruz Biotechnology), GLI Family Zinc Finger 2 (Gli2) (GWB-CE7858, Genway Biotech), Smo (ab236465, Abcam), ki67 (ab16667, Abcam) and CyclinD1 (ab134175, Abcam). Secondary antibodies were HRP-conjugated anti-rabbit (K4003, DAKO) and anti-mouse (K4001, DAKO) antibodies. Blocking and chromogenic detection was performed using the DAKO Envision System with DAB substrate (DAKO Corporation) according to the manufacturer’s protocol. Tissue sections were counterstained with Hematoxylin Gill Nº1 (Sigma Aldrich). The specific staining was visualized and ten images per liver were acquired using a microscope equipped with a digital camera. Percentage of positive area or positive cells per field were quantified using ImageJ software.

**Genomic DNA extraction**

Genomic DNA was isolated and purified from liver tissue samples using DNeasy mini kit (Qiagen). All DNA samples were tested for purity and integrity using a Nanodrop 2000 spectrophotometer. Genomic DNA was used for performing Telomere Length and Mitochondrial DNA qPCR assays.

**Telomere length measurement by quantitative PCR**

Telomere length measurement was performed using a quantitative PCR-based method following manufacturer’s instructions (M8908, ScienCell). The relative telomere length was calculated as the ratio of telomere repeats to a single-copy gene provided by the kit (T/S ratio).

**Mitochondrial DNA determination**

Mitochondrial DNA was determined using a quantitative PCR-based method. Mitochondrial DNA genes NADH dehydrogenase 1 (MT-ND1) and ribosomal protein S16 (MT-S16) were detected using specific primers and results were normalized to a single-copy gene (S9). The sequences of primers used in this study are listed (Table S1).

**Mitochondrial respiration**

Primary hepatocytes isolated from 4 unique healthy control mice were treated for 24h with vehicle (DMSO) or 5 μM Cyclopamine, a direct antagonist of Smo. Mitochondrial bioenergetics were assessed using a Seahorse XCFa analyser (Agilent Technologies) after the incubation time. Mitochondrial respiration in a coupled state (10 μg/well) was measured in mitochondrial assay solution (MAS; 220 mM mannitol, 70 mM sucrose, 10 mM KH2PO4, 5 mM MgCl2, 2 mM HEPES, 1 mM EGTA and 0.2% (w/v) fatty acid-free BSA, pH 7.2 at 37ºC) containing succinate as a substrate (10 mM) and rotenone (1 μM). State 3 respiration (phosphorylating respiration) was triggered via the injection of 4 mM adenosine diphosphate (ADP). State of respiration was assessed by the addition of 0.5 μg/mL oligomycin, while maximal uncoupler-stimulated respiration was observed following the injection of 0.3 μM carbonyl cyanide 4-(trifluoromethoxy)phenylhydrazone (FCCP). Antimycin A (1 μM), a Complex III inhibitor, and rotenone (1 μM), a Complex I inhibitor, were added at the end of the experiment to inhibit mitochondrial respiration, as described (1). Every sample was analysed in triplicate. The Seahorse XF Wave software was used to group the respiration data from separate mice into a single representative curve and data was normalized to mitochondrial protein content.

**Supplementary table 1**

| Gene symbol | Primer forward | Primer reverse |
| --- | --- | --- |
| Mm S9 | GGGCCTGAAGATTGAGGATT | CGGGCATGGTGAATAGATTT |
| Mm FoxM1 | TGATAGCCTCAGCAAGATCC | CTATCGCAGCTAACCGATGA |
| Mm CyclinD1 | TAGGCCCTCAGCCTCACTC | CCACCCCTGGGATAAAGCAC |
| Mm Ihh | CTGGCGCGCTTAGCAGTGGA | GCCACACGCTCCCCGTTCTC |
| Mm Gli2 | GCCTTCACCCACCTTCTT | TTCTGCTTGTGGTTGGCATCAT |
| Mm Smo | CAGTCAGGAATGGGCTTCTT | GCTGCCACTTCTATGACTTCT |
| Mm Mt-S16 | CCGCAAGGGAAAGATGAAAGA | TCGTTTGGTTTCGGGGTT |
| Mm Mt-ND1 | CTAGCAGAAACAAACCGGGC | CCGGCTGCGTATTCTACGTT |

**Supplementary table 2**

Top 20 downregulated gene sets in old compared to young hepatocytes

| NAME | NES | NOM p-val | FDR q-val |
| --- | --- | --- | --- |
| HALLMARK_MITOTIC_SPINDLE | -1.96 | 0.00 | 0.00 |
| HALLMARK_UV_RESPONSE_DN | -1.75 | 0.00 | 0.00 |
| HALLMARK_TGF_BETA_SIGNALING | -1.56 | 0.01 | 0.03 |
| HALLMARK_HEDGEHOG_SIGNALING | -1.56 | 0.02 | 0.02 |
| HALLMARK_TNFA_SIGNALING_VIA_NFKB | -1.46 | 0.00 | 0.06 |
| HALLMARK_NOTCH_SIGNALING | -1.45 | 0.05 | 0.06 |
| HALLMARK_KRAS_SIGNALING_DN | -1.41 | 0.03 | 0.07 |
| HALLMARK_G2M_CHECKPOINT | -1.24 | 0.07 | 0.30 |
| HALLMARK_ANDROGEN_RESPONSE | -1.24 | 0.13 | 0.27 |
| HALLMARK_ESTROGEN_RESPONSE_EARLY | -1.21 | 0.11 | 0.30 |
| HALLMARK_IL2_STAT5_SIGNALING | -1.19 | 0.14 | 0.32 |
| HALLMARK_APICAL_JUNCTION | -1.16 | 0.19 | 0.38 |
| HALLMARK_SPERMATOGENESIS | -1.11 | 0.29 | 0.45 |
| HALLMARK_PI3K_AKT_MTOR_SIGNALING | -1.03 | 0.42 | 0.65 |
| HALLMARK_HEME_METABOLISM | -1.01 | 0.44 | 0.66 |
| HALLMARK_WNT_BETA_CATENIN_SIGNALING | -1.00 | 0.47 | 0.63 |
| HALLMARK_HYPOXIA | -0.98 | 0.49 | 0.65 |
| HALLMARK_APICAL_SURFACE | -0.90 | 0.63 | 0.83 |
| HALLMARK_P53_PATHWAY | -0.87 | 0.77 | 0.84 |

**Supplementary table 3**

Top 20 upregulated gene sets in old compared to young hepatocytes

| NAME | NES | NOM p-val | FDR q-val |
| --- | --- | --- | --- |
| HALLMARK_OXIDATIVE_PHOSPHORYLATION | 2.62 | 0.00 | 0.00 |
| HALLMARK_MYC_TARGETS_V1 | 2.49 | 0.00 | 0.00 |
| HALLMARK_FATTY_ACID_METABOLISM | 2.21 | 0.00 | 0.00 |
| HALLMARK_MTORC1_SIGNALING | 2.03 | 0.00 | 0.00 |
| HALLMARK_DNA_REPAIR | 2.02 | 0.00 | 0.00 |
| HALLMARK_PEROXISOME | 1.88 | 0.00 | 0.00 |
| HALLMARK_ALLOGRAFT_REJECTION | 1.83 | 0.00 | 0.00 |
| HALLMARK_INTERFERON_ALPHA_RESPONSE | 1.80 | 0.00 | 0.00 |
| HALLMARK_MYC_TARGETS_V2 | 1.73 | 0.00 | 0.00 |
| HALLMARK_GLYCOLYSIS | 1.72 | 0.00 | 0.00 |
| HALLMARK_COAGULATION | 1.68 | 0.00 | 0.00 |
| HALLMARK_REACTIVE_OXYGEN_SPECIES_PATHWAY | 1.65 | 0.01 | 0.00 |
| HALLMARK_E2F_TARGETS | 1.65 | 0.00 | 0.00 |
| HALLMARK_XENOBIOTIC_METABOLISM | 1.64 | 0.00 | 0.00 |
| HALLMARK_ADIPOGENESIS | 1.62 | 0.00 | 0.01 |
| HALLMARK_INTERFERON_GAMMA_RESPONSE | 1.55 | 0.00 | 0.01 |
| HALLMARK_APOPTOSIS | 1.40 | 0.00 | 0.04 |
| HALLMARK_KRAS_SIGNALING_UP | 1.25 | 0.04 | 0.12 |
| HALLMARK_CHOLESTEROL_HOMEOSTASIS | 1.20 | 0.11 | 0.17 |

**Supplementary table 4**

Top 20 downregulated gene sets in old regenerating compared to young hepatocytes

| NAME | NES | NOM p-val | FDR q-val |
| --- | --- | --- | --- |
| HALLMARK_MITOTIC_SPINDLE | -1.96 | 0.00 | 0.00 |
| HALLMARK_UV_RESPONSE_DN | -1.88 | 0.00 | 0.00 |
| HALLMARK_HEDGEHOG_SIGNALING | -1.68 | 0.00 | 0.01 |
| HALLMARK_KRAS_SIGNALING_DN | -1.63 | 0.00 | 0.01 |
| HALLMARK_TGF_BETA_SIGNALING | -1.57 | 0.01 | 0.02 |
| HALLMARK_APICAL_JUNCTION | -1.41 | 0.01 | 0.09 |
| HALLMARK_BILE_ACID_METABOLISM | -1.40 | 0.01 | 0.08 |
| HALLMARK_WNT_BETA_CATENIN_SIGNALING | -1.39 | 0.05 | 0.08 |
| HALLMARK_IL2_STAT5_SIGNALING | -1.37 | 0.01 | 0.08 |
| HALLMARK_APICAL_SURFACE | -1.26 | 0.12 | 0.16 |
| HALLMARK_PANCREAS_BETA_CELLS | -1.26 | 0.16 | 0.15 |
| HALLMARK_MYOGENESIS | -1.24 | 0.04 | 0.16 |
| HALLMARK_NOTCH_SIGNALING | -1.23 | 0.18 | 0.16 |
| HALLMARK_INFLAMMATORY_RESPONSE | -1.17 | 0.13 | 0.24 |
| HALLMARK_ESTROGEN_RESPONSE_EARLY | -1.10 | 0.18 | 0.36 |
| HALLMARK_KRAS_SIGNALING_UP | -1.04 | 0.35 | 0.48 |
| HALLMARK_HEME_METABOLISM | -1.03 | 0.33 | 0.48 |
| HALLMARK_ANGIOGENESIS | -1.02 | 0.39 | 0.47 |
| HALLMARK_TNFA_SIGNALING_VIA_NFKB | -0.99 | 0.48 | 0.54 |
| HALLMARK_COMPLEMENT | -0.93 | 0.67 | 0.70 |
| HALLMARK_ANDROGEN_RESPONSE | -0.92 | 0.63 | 0.68 |

**Supplementary table 5**

Top 20 upregulated gene sets in old regenerating compared to young hepatocytes

| NAME | NES | NOM p-val | FDR q-val |
| --- | --- | --- | --- |
| HALLMARK_OXIDATIVE_PHOSPHORYLATION | 2.62 | 0.00 | 0.00 |
| HALLMARK_E2F_TARGETS | 2.48 | 0.00 | 0.00 |
| HALLMARK_G2M_CHECKPOINT | 2.36 | 0.00 | 0.00 |
| HALLMARK_MYC_TARGETS_V1 | 2.35 | 0.00 | 0.00 |
| HALLMARK_DNA_REPAIR | 2.08 | 0.00 | 0.00 |
| HALLMARK_OXIDATIVE_PHOSPHORYLATION | 2.07 | 0.00 | 0.00 |
| HALLMARK_MTORC1_SIGNALING | 1.94 | 0.00 | 0.00 |
| HALLMARK_MYC_TARGETS_V2 | 1.89 | 0.00 | 0.00 |
| HALLMARK_GLYCOLYSIS | 1.77 | 0.00 | 0.00 |
| HALLMARK_MITOTIC_SPINDLE | 1.77 | 0.00 | 0.00 |
| HALLMARK_SPERMATOGENESIS | 1.77 | 0.00 | 0.00 |
| HALLMARK_UNFOLDED_PROTEIN_RESPONSE | 1.63 | 0.00 | 0.00 |
| HALLMARK_FATTY_ACID_METABOLISM | 1.57 | 0.00 | 0.01 |
| HALLMARK_APOPTOSIS | 1.52 | 0.01 | 0.01 |
| HALLMARK_ESTROGEN_RESPONSE_LATE | 1.43 | 0.01 | 0.03 |
| HALLMARK_REACTIVE_OXYGEN_SPECIES_PATHWAY | 1.42 | 0.04 | 0.04 |
| HALLMARK_UV_RESPONSE_UP | 1.33 | 0.03 | 0.08 |
| HALLMARK_PEROXISOME | 1.33 | 0.05 | 0.08 |
| HALLMARK_PROTEIN_SECRETION | 1.20 | 0.16 | 0.22 |
| HALLMARK_ADIPOGENESIS | 1.18 | 0.13 | 0.24 |
| HALLMARK_ALLOGRAFT_REJECTION | 1.18 | 0.15 | 0.24 |

**Supplementary table 6**

Top 20 downregulated gene sets in Smo (-) compared to Smo (+) hepatocytes

| NAME | NES | NOM p-val | FDR q-val |
| --- | --- | --- | --- |
| HALLMARK_ALLOGRAFT_REJECTION | -2.28 | 0.00 | 0.00 |
| HALLMARK_COAGULATION | -1.86 | 0.00 | 0.00 |
| HALLMARK_ANGIOGENESIS | -1.81 | 0.00 | 0.00 |
| HALLMARK_KRAS_SIGNALING_UP | -1.63 | 0.00 | 0.02 |
| HALLMARK_COMPLEMENT | -1.57 | 0.00 | 0.02 |
| HALLMARK_KRAS_SIGNALING_DN | -1.55 | 0.02 | 0.02 |
| HALLMARK_INTERFERON_GAMMA_RESPONSE | -1.52 | 0.00 | 0.03 |
| HALLMARK_INFLAMMATORY_RESPONSE | -1.52 | 0.00 | 0.03 |
| HALLMARK_EPITHELIAL_MESENCHYMAL_TRANSITION | -1.49 | 0.00 | 0.03 |
| HALLMARK_INTERFERON_ALPHA_RESPONSE | -1.43 | 0.01 | 0.04 |
| HALLMARK_IL6_JAK_STAT3_SIGNALING | -1.43 | 0.03 | 0.04 |
| HALLMARK_IL2_STAT5_SIGNALING | -1.34 | 0.02 | 0.08 |
| HALLMARK_APICAL_JUNCTION | -1.31 | 0.03 | 0.09 |
| HALLMARK_WNT_BETA_CATENIN_SIGNALING | -1.22 | 0.19 | 0.16 |
| HALLMARK_MYOGENESIS | -1.07 | 0.28 | 0.42 |
| HALLMARK_HEDGEHOG_SIGNALING | -1.03 | 0.41 | 0.47 |
| HALLMARK_APOPTOSIS | -1.03 | 0.38 | 0.44 |
| HALLMARK_UV_RESPONSE_UP | -0.98 | 0.50 | 0.55 |
| HALLMARK_NOTCH_SIGNALING | -0.90 | 0.60 | 0.72 |

**Supplementary table 7**

Top 20 upregulated gene sets in Smo (-) compared to Smo (+) hepatocytes

| NAME | NES | NOM p-val | FDR q-val |
| --- | --- | --- | --- |
| HALLMARK_FATTY_ACID_METABOLISM | 2.41 | 0.00 | 0.00 |
| HALLMARK_OXIDATIVE_PHOSPHORYLATION | 2.37 | 0.00 | 0.00 |
| HALLMARK_MYC_TARGETS_V1 | 2.13 | 0.00 | 0.00 |
| HALLMARK_ADIPOGENESIS | 2.04 | 0.00 | 0.00 |
| HALLMARK_BILE_ACID_METABOLISM | 2.03 | 0.00 | 0.00 |
| HALLMARK_MTORC1_SIGNALING | 1.94 | 0.00 | 0.00 |
| HALLMARK_HYPOXIA | 1.81 | 0.00 | 0.00 |
| HALLMARK_PEROXISOME | 1.80 | 0.00 | 0.00 |
| HALLMARK_XENOBIOTIC_METABOLISM | 1.78 | 0.00 | 0.00 |
| HALLMARK_PROTEIN_SECRETION | 1.72 | 0.00 | 0.00 |
| HALLMARK_CHOLESTEROL_HOMEOSTASIS | 1.69 | 0.00 | 0.00 |
| HALLMARK_REACTIVE_OXYGEN_SPECIES_PATHWAY | 1.64 | 0.00 | 0.01 |
| HALLMARK_GLYCOLYSIS | 1.57 | 0.00 | 0.01 |
| HALLMARK_ANDROGEN_RESPONSE | 1.50 | 0.01 | 0.02 |
| HALLMARK_TNFA_SIGNALING_VIA_NFKB | 1.43 | 0.01 | 0.05 |
| HALLMARK_PANCREAS_BETA_CELLS | 1.40 | 0.12 | 0.06 |
| HALLMARK_PI3K_AKT_MTOR_SIGNALING | 1.39 | 0.03 | 0.06 |
| HALLMARK_UNFOLDED_PROTEIN_RESPONSE | 1.37 | 0.04 | 0.06 |
| HALLMARK_HEME_METABOLISM | 1.37 | 0.02 | 0.06 |

**Supplementary Figure 1**

**
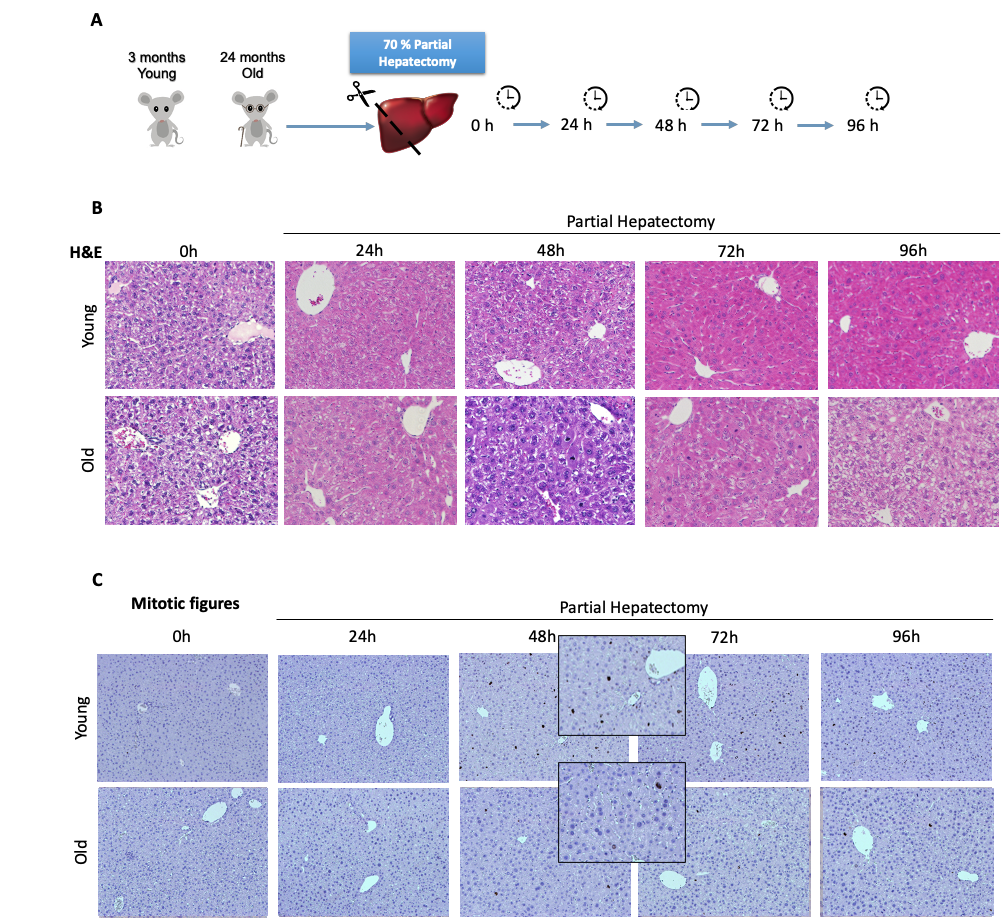
**

**Supplementary Figure 1. Liver regeneration is impaired with aging.** Partial hepatectomy (70%) was performed in young (3 month-old) and old (24 month-old) mice. Liver tissue and hepatocytes samples were collected at 0h and 24, 48, 72 and 96h post-PH (A). Hepatic architecture from control and 24h, 48h, 72h and 96h following PH in young and old mice. Representative images of hematolxylin & eosin staining in liver tissue (100X) (B). Representative micrographs of P-Histone 3 immunohistochemistry in liver sections from control and 24h, 48h, 72h and 96h following PH in young and old mice (100X magnification) (C). Results shown as MEAN +/- SEM (n=6 mice/group/time, *p<0.05,***p<0.001).

**Supplementary Figure 2**

**
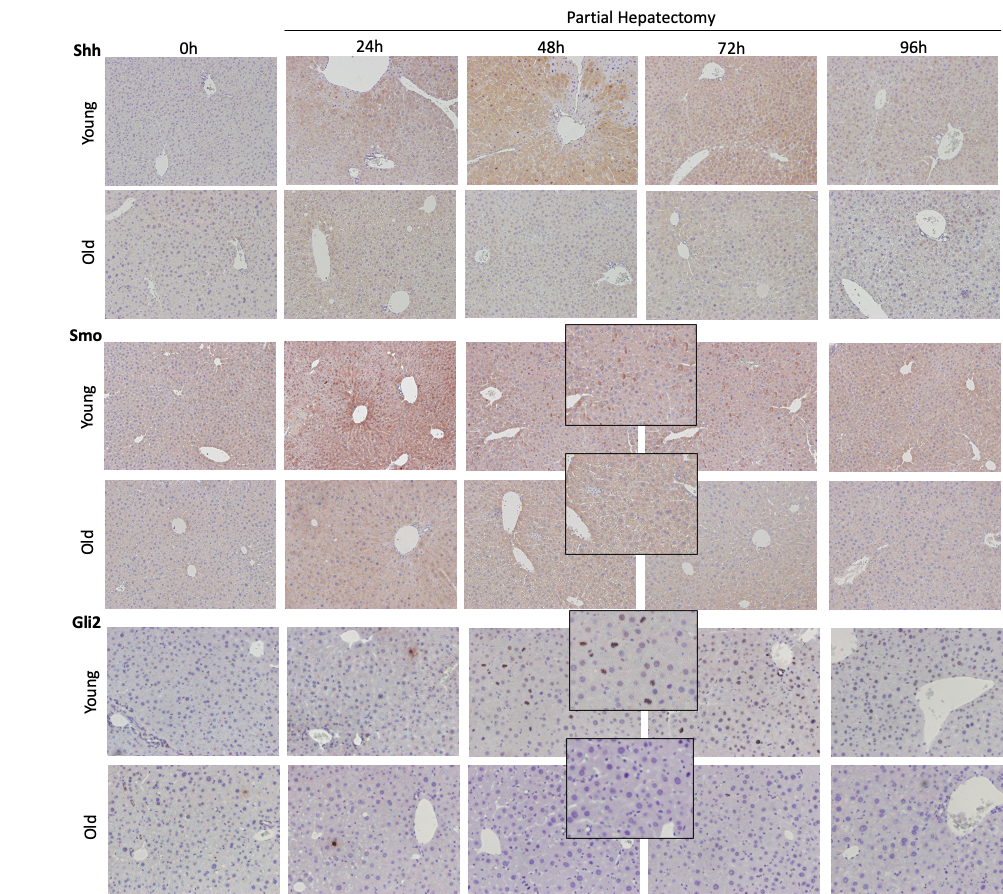
**

**Supplementary Figure 2. Hedgehog signaling pathway proteins are downregulated in old regenerating hepatocytes.** Representative micrographs of Sonic Hedgehog (Shh), Smoothened (Smo) and GLI Family Zinc Finger 2 (Gli2) immunohistochemistry in liver sections from control and 24h, 48h, 72h and 96h following PH in young and old mice (100X magnification, n=6 mice/group/time).

**Supplementary Figure 3**

**
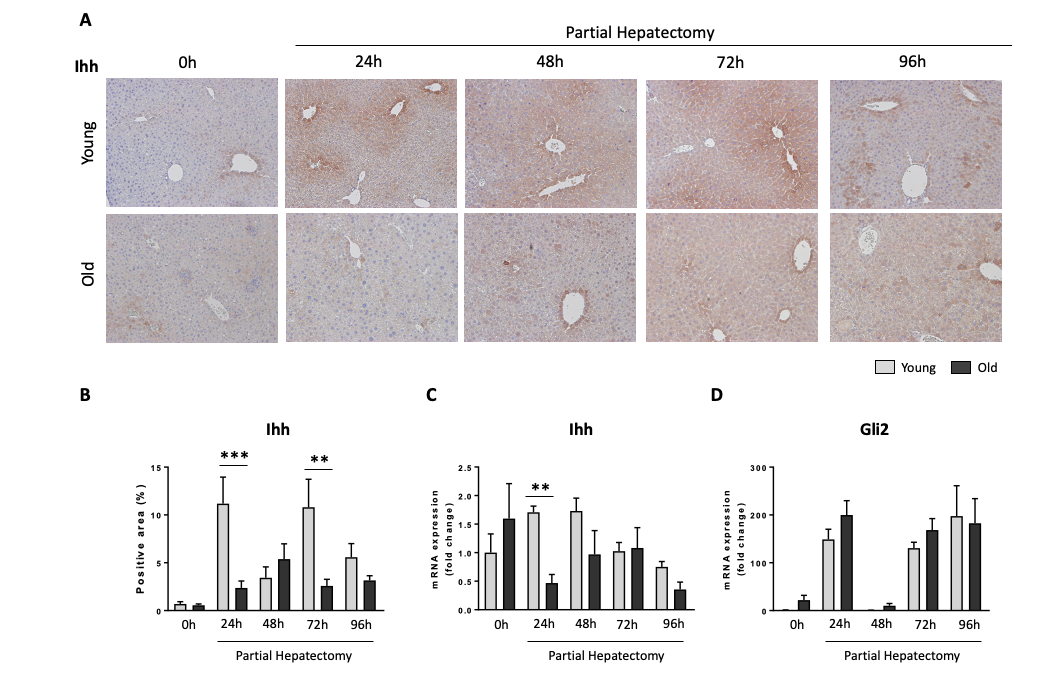
**

**Supplementary Figure 3. Indian Hedgehog ligand expression is supressed in old mice after partial hepatectomy.** Representative micrographs (A) and quantitative analysis (B) of Indian Hedgehog immunohistochemistry in liver sections from control and 24h, 48h, 72h and 96h following PH in young and aged mice (100X magnification). Indian Hedgehog (Ihh) (C) and Gli2 (D) hepatocyte mRNA expression. Results shown as MEAN +/- SEM (n=6 mice/group/time, **p<0.01,***p<0.001).

**Supplementary Figure 4**


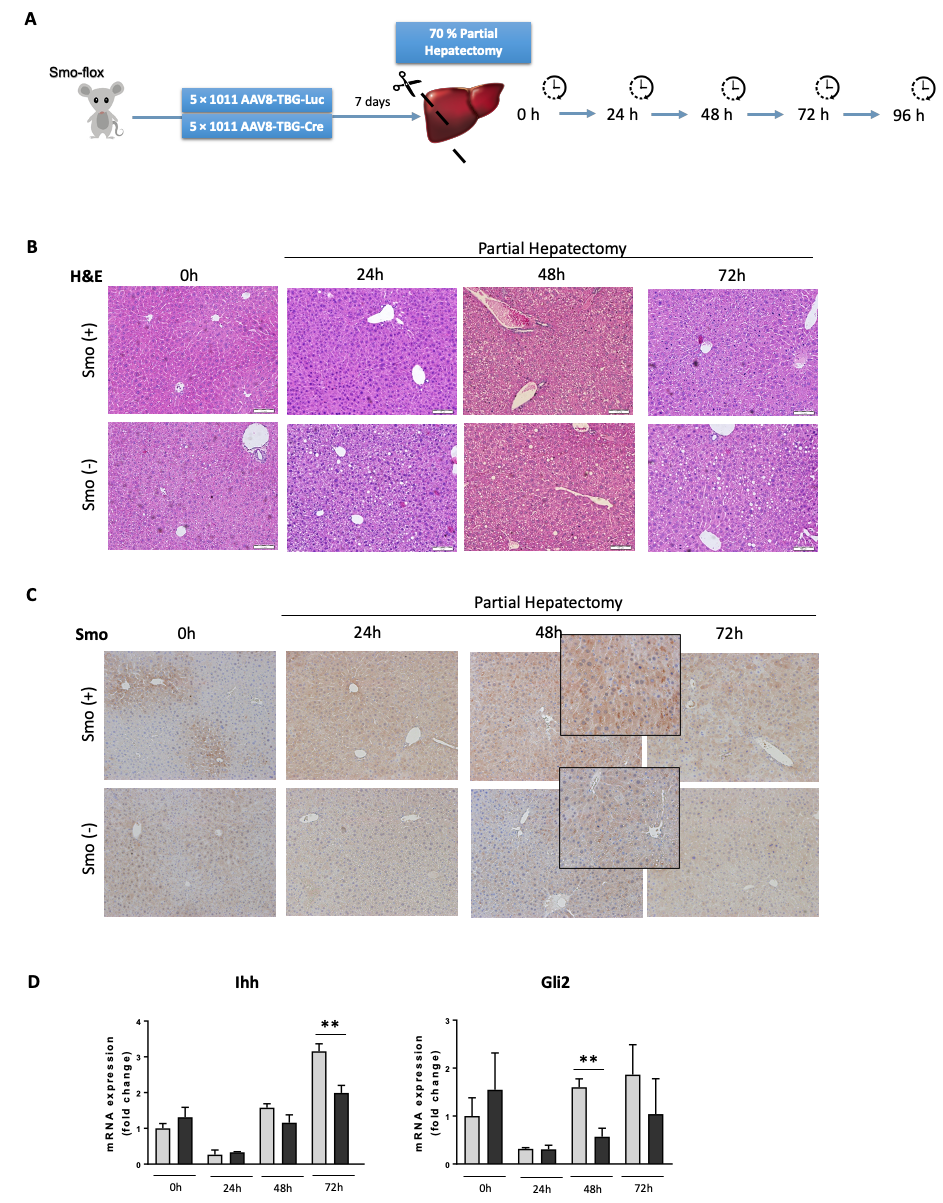


**Supplementary Figure 4. Smoothened deletion in hepatocytes effectively inhibits Hedgehog signaling pathway.** Smo floxed mice were injected with AAV8-TBG-Cre recombinase (Smo (-) or AAV8-TBG-Luciferase (Smo (+). Partial hepatecetomy (PH) was performed seven days later. Liver and hepatocytes were harvested before or 24, 48, 72 and 96h post-PH (A). Hepatic architecture from control and 24h, 48h and 72h following PH in AAV8-TBG-Cre recombinase (Smo (-)) or AAV8-TBG-Luciferase (Smo (+)) mice. Representative images of hematolxylin & eosin staining in liver tissue (100X) (B). Representative micrographs of Smo immunohistochemistry in liver sections (100X) (C). Indian Hedgehog (Ihh) and Gli2 hepatocyte mRNA expression (D). Results shown as MEAN +/- SEM (n=5 mice/group/time, **p<0.01).

**Supplementary Figure 5**


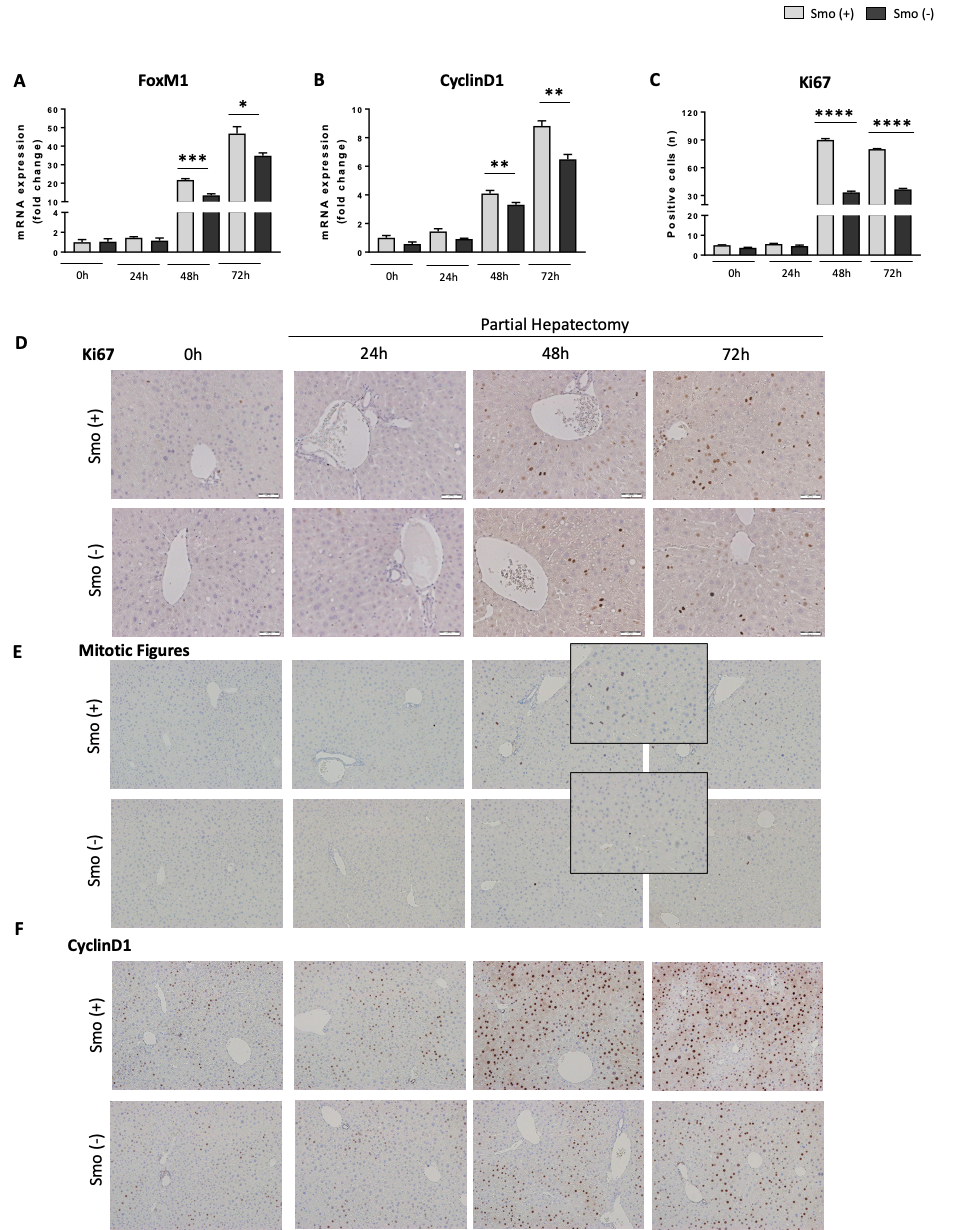


**Supplementary Figure 5. Smoothened deletion in hepatocytes inhibits hepatocyte cell cycle progression after partial hepatectomy.** *Smo* floxed mice were injected with AAV8-TBG-Cre recombinase (Smo (-) or AAV8-TBG-Luciferase (Smo (+). Partial hepatectomy was performed seven days later. Liver and hepatocytes were harvested before or 24, 48 and 72 h post-PH. FoxM1 (A) and Cyclin D1 (B) hepatocyte mRNA expression. Quantitative analysis and representative micrographs of Ki67 (C and D), mitotic figures (E) and Cyclin D1 (F) immunohistochemistry in liver sections (100X magnification). Results shown as MEAN +/- SEM (n=5 mice/group/time,*p<0.05, **p<0.01, ***p<0.001, ****p<0.0001).

**Supplementary Figure 6**

**
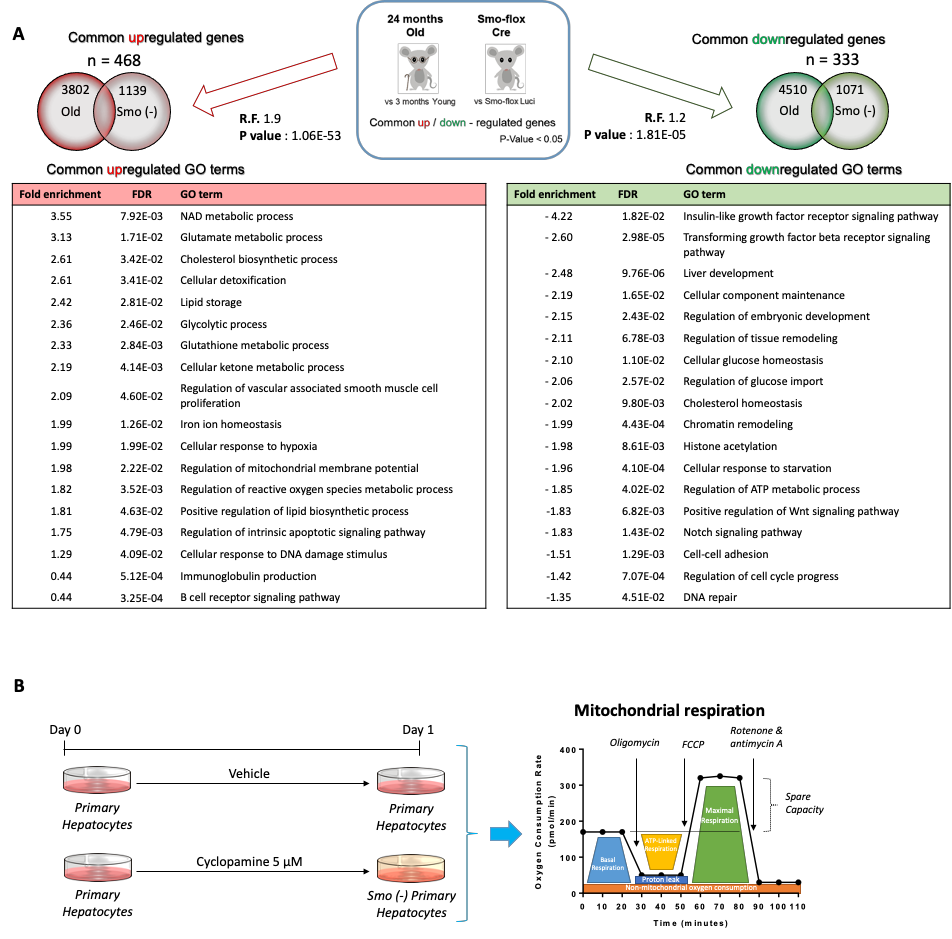
**

**Supplementary Figure 6. Smoothened deficient young hepatocytes resemble old hepatocytes.** RNAseq was performed in hepatocytes isolated before PH (time 0h) from control in young (n = 3) and old (n = 3) mice and Smo (+) (n = 1) and Smo (-) (n = 2). (A) Old and Smo (-) commonly and signicanlty upregulated (*left*) and downregulated (*right*) genes were analyzed using Gene Ontology (GO) and representative GO terms are shown in corresponding tables. (B) Primary hepatocytes isolated from healthy control mice were treated for 24h with vehicle (DMSO) or 5 μM Cyclopamine (n = 4 mice/group). Mitochondrial bioenergetics were assessed using a Seahorse XF96 analyzer after the incubation time.

**Supplementary References**

1. Hartman JH, Smith LL, Gordon KL, Laranjeiro R, Driscoll M, Sherwood DR, et al. Swimming Exercise and Transient Food Deprivation in Caenorhabditis elegans Promote Mitochondrial Maintenance and Protect Against Chemical-Induced Mitotoxicity. Sci Rep. 2018;8(1):8359.
